# Emergence of pandrug-resistant carbapenemase-producing *Enterobacterales* in dogs and cats: a cross-sectional study in Egypt

**DOI:** 10.3389/fcimb.2024.1318585

**Published:** 2024-03-18

**Authors:** Yasmine H. Tartor, Ahmed M. Ammar, Adel Abdelkhalek, Khlood A. Hassan, Asmaa Shaker, Shimaa S. Elnahriry, Omid Nekouei, Ibrahim Elsohaby

**Affiliations:** ^1^ Department of Microbiology, Faculty of Veterinary Medicine, Zagazig University, Zagazig, Egypt; ^2^ Faculty of Veterinary Medicine, Badr University, Cairo, Egypt; ^3^ Faculty of Veterinary Medicine, Zagazig University, Zagazig, Egypt; ^4^ Department of Microbiology, Veterinary Hospital, Faculty of Veterinary Medicine, University of Sadat City, Sadat City, Egypt; ^5^ Department of Bacteriology, Mycology and Immunology, Faculty of Veterinary Medicine, University of Sadat City, Menofia, Egypt; ^6^ Department of Infectious Diseases and Public Health, Jockey Club College of Veterinary Medicine and Life Sciences, City University of Hong Kong, Hong Kong, Hong Kong SAR, China; ^7^ Centre for Applied One Health Research and Policy Advice (OHRP), City University of Hong Kong, Hong Kong, Hong Kong SAR, China; ^8^ Department of Animal Medicine, Faculty of Veterinary Medicine, Zagazig University, Zagazig, Egypt

**Keywords:** carbapenem-resistant *Enterobacterales*, pandrug-resistant *K. pneumoniae*, Carba NP test, NG-Test CARBA 5, carbapenemase detection methods, healthy and diseased pets

## Abstract

One of the most important emerging health problems is the increasing role of animals in the rapid global rise in resistance to last-resort antibiotics, such as carbapenems. However, there is limited information on the role of pet animals in harboring and spreading pandrug-resistant (PDR) carbapenemase-producing *Enterobacterales* (CPE), especially in Egypt. This cross-sectional study was conducted to screen for CPE in healthy and diseased pets using phenotypic and molecular methods and the NG-Test CARBA 5 immunochromatographic assay. Rectal swabs were collected from 62 dogs and 48 cats, incubated overnight in tryptic soy broth containing 10 μg of meropenem disc and subsequently cultured on MacConkey agar supplemented with meropenem (1 mg/L). Sixty-six isolates (60.6%), including 56 *Klebsiella pneumoniae*, seven *Escherichia coli*, and three *K. oxytoca* isolates, were confirmed to be carbapenem-resistant *Enterobacterales* (CRE) by the disc diffusion method, broth microdilution test, CNPt-direct, and PCR assay targeting carbapenemase genes. Forty-three (65.2%) dogs and 23 (34.8%) cats carried CPE. Of these, 35 (70.0%) were healthy (including 27 dogs and 8 cats) and 31 (52.5%) were diseased (including 16 dogs and 15 cats). *bla*_OXA-181_ was the most common gene detected (42/66, 63.6%), followed by *bla*_IMP_ (40/66, 60.6%), *bla*_OXA−48−like_ (29/66, 43.9%), *bla*_KPC_ and *bla*_VIM_ (20/66, 30.3% each), and *bla*_NDM_ (17/66, 25.8%). The identified genotypes were *bla*_KPC-2_, *bla*_IMP-1_, *bla*_VIM-1_, *bla*_NDM-1_, and *bla*_NDM-5_. The CARBA 5 assay showed higher sensitivity and specificity for the detection of NDM, OXA and KPC than that for VIM and IMP genes. Antimicrobial resistance profiles of CRE isolates revealed 20 PDR, 30 extensively drug-resistant (XDR), and 16 multidrug-resistant (MDR) phenotypes. This study provides evidence of colonization with PDR CPE in dogs and cats. To manage the infection or colonization of pets in veterinary clinical settings, extended surveillance systems should be considered, and the use of critical antibiotics should be strictly controlled.

## 1 Introduction

Antimicrobial resistance poses a serious global threat to both human and animal health, particularly the increasing resistance to carbapenems, which are the last resort *β*‐lactam antimicrobial used to treat multidrug-resistant (MDR) Gram-negative bacterial (GNB) infections (Logan and Weinstein, 2017; Silva et al., 2022). The emergence and global dissemination of carbapenem-resistant *Enterobacterales* (CRE) strains are significant threats to public health due to their rapid spread in various environments, their association with high morbidity and mortality, and the limited therapeutic options available for these infections (Chavda et al., 2016; Logan and Weinstein, 2017). CREs are among the most challenging MDR pathogens that have emerged in the clinical setting, because they have lost susceptibility to nearly all *β*‐lactam antibiotics and have developed co‐resistance to various critically important classes of antimicrobial agents (Magiorakos et al., 2012; Patel and Bonomo, 2013). Carbapenemase production is the most pervasive and epidemiologically significant resistance mechanism in GNB, with the majority of CRE isolates being from clinical sources attributed to carbapenemase-producing *Enterobacterales* (CPE) (Temkin et al., 2014). Several distinct carbapenemases, including class A serine *β*-lactamases (most commonly KPC-type enzymes), class B metallo-*β*-lactamases (NDM-, VIM- and IMP-type enzymes), and class D serine *β*-lactamases (predominantly OXA-48 and related enzymes), can be found among CPEs (Stojanoski et al., 2021). Different carbapenemase classes exhibit distinct functional characteristics, which can be important for phenotypic detection (EARS-NET, E, 2017).

Early and accurate detection of CRE via ongoing surveillance is crucial for effective antimicrobial therapy management and control measures (Logan and Weinstein, 2017; Bayraktar et al., 2019). Clinical laboratories are expected to differentiate between various carbapenemase types due to the distinctive characteristics of these enzymes and the limitations of available antimicrobial agents (Zhang et al., 2022). To achieve this goal, it is essential to introduce rapid and accurate methods for CPE detection (Zhou et al., 2018). In clinical laboratories, the identification and differentiation of carbapenemases from cultured isolates typically involve antimicrobial susceptibility testing followed by phenotypic carbapenemase production detection (e.g., Carbapenemase Nordmann-Poirel [Carba NP] or the modified carbapenem inactivation method [mCIM]) and/or molecular detection of specific carbapenemase genes (Van Der Zee et al., 2014; Tamma and Simner, 2018).

Although phenotypic CRE detection methods with enhanced performance characteristics and rapid molecular approaches have been significantly developed over the past decade, these methods still have several limitations, such as complexity, cost, turnaround time, unsuitability for common clinical laboratories, and an inability to detect all carbapenemase variants (Tamma and Simner, 2018; Zhang et al., 2022). The NG-Test Carba 5, on the other hand, is a simple and rapid immunoassay for the detection and differentiation of the five most common carbapenemase families (KPC, VIM, NDM, IMP, and OXA-48-like) directly from bacterial colonies (Diagnostics, H, 2019; Jenkins et al., 2020; Zhang et al., 2022). The NG-Test Carba 5 has shown inclusiveness with at least 15 different confirmed variants, including OXA-163, OXA-181, and OXA-232, within the OXA-48-like family (Diagnostics, H, 2019; Jenkins et al., 2020). To date, there have been no reports of such an immunochromatographic detection assay being used for CRE in animal‐derived samples.

Carbapenems are classified as category A (“Avoid”) antibiotics for animal use by the European Medicine Agency, indicating their prohibition in veterinary medicine in the European Union, except for certain clinical cases in companion animals (EMA, 2019). Although carbapenems are not frequently used in veterinary medicine, there are approximately 26 reports of CRE and CPE infection or colonization in dogs and cats worldwide (Silva et al., 2022). Companion animals frequently interact with humans, which creates favorable conditions for transmission of CPE (Nigg et al., 2019; Ramadan et al., 2020; Khalifa et al., 2021). The detection of CPE in companion animals has sparked public health concerns because CPE could potentially serve as a reservoir of carbapenem resistance determinants and facilitate the spread of CRE (Pomba et al., 2017).

In Egypt, there is limited information available regarding the frequency of CRE and CPE in pet animals. Therefore, this study was conducted to (i) detect and characterize (phenotypically and genotypically) CRE from both healthy and diseased dogs and cats in Egypt, and (ii) assess the diagnostic performance of the CNP-direct test and the NG-Test CARBA 5 for detection of CRE in clinical isolates.

## 2 Materials and methods

### 2.1 Animals and sampling

**Figure 1:**
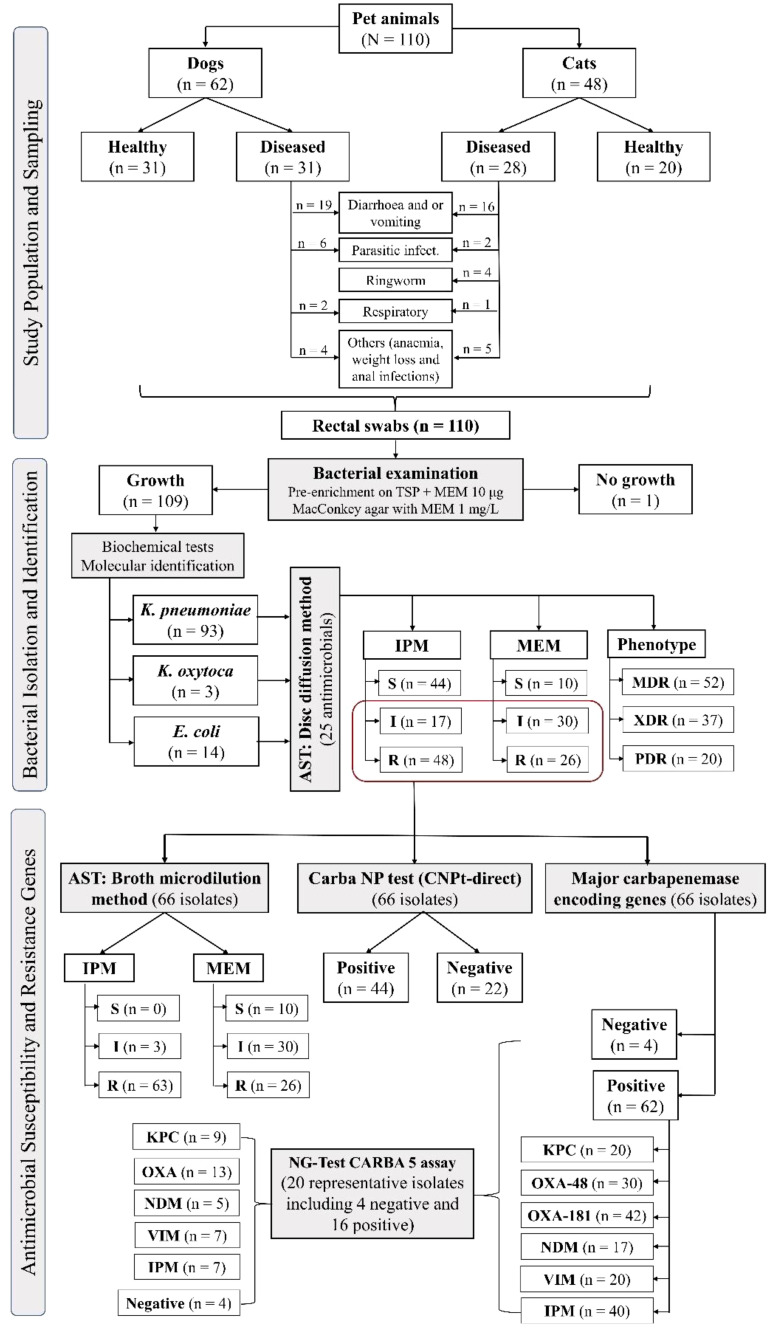
Schematic flowchart of the study design, including sampling and bacterial isolation, identification, and antimicrobial susceptibility.

The study was conducted between November 2021 and December 2022 and included 62 dogs and 48 cats admitted to three different veterinary clinics in Cairo, Dakahlia, and Sharkia Governorates, Egypt. Animals (n = 110) were categorized into two groups based on the purpose of their visit to the veterinary clinics: (a) apparently healthy animals (51 dogs and cats): admitted for vaccination; (b) diseased animals (59 dogs and cats): admitted with one or more of the following clinical signs: diarrhea, vomiting, respiratory signs, parasitic manifestation, ringworm, otitis, anemia, or weight loss (**Figure 1**). The sampled pets consisted of 42.7% males and 46.4% females, with a median age of 7 months (range 1.5 to 85 months) (**Supplementary Table S1**). The study population primarily consisted of domestic pets (94.6%), with a small percentage (5.5%) being stray animals. Additionally, more than 50% of the samples were collected from Cairo, and 78.2% of the animals had not received antimicrobial treatment within 4 weeks.

Rectal swabs were collected and immediately transported in an icebox to the laboratory for bacteriological examination. Animal participation was voluntary and the animal owners were asked to provide written informed consent prior to sampling.

### 2.2 Culture screening method for detecting CRE

Rectal swabs were placed in 5 ml of tryptic soy broth (HiMedia, Mumbai, India) supplemented with a 10 μg meropenem disc (Oxoid, Cambridge, UK) for overnight selective enrichment at 37°C (CDC, 2009). A loopful from the enrichment broth was streaked onto MacConkey agar supplemented with meropenem (1 mg/L) (Sigma-Aldrich, Seelze, Germany) and then incubated at 37°C for 24 h. Subsequently, the agar plates were examined for presumptive carbapenemase-producing *Enterobacterales* (CPE), which were identified based on their growth as either lactose-fermenting or lactose-nonfermenting colonies (Marques et al., 2019).

### 2.3 Identification of CRE

The suspected CPE colonies were subcultured from the screening plates onto MacConkey plates supplemented with meropenem to isolate pure cultures. Identification of the isolates was performed based on morphological characteristics after Gram staining, culture on eosin methylene blue agar media (Oxoid, Cambridge, UK), and biochemical tests including indole, methyl red, Voges–Proskauer, urease and citrate tests (Quinn et al., 1994). Additionally, the species identities were confirmed using the species-specific primers listed in **Supplementary Table S2**.

### 2.4 Antimicrobial susceptibility testing

#### 2.4.1 Disc diffusion method

The susceptibility patterns of *Enterobacterales* isolates to various antimicrobial agents were determined using the Kirby–Bauer disc diffusion method following the Clinical and Laboratory Standards Institute (CLSI) guidelines and interpretative criteria (CLSI, 2022). Each isolate was tested against 25 antibiotics representing 15 antimicrobial groups including: amoxicillin-clavulanic acid (AMC, 30 μg), ampicillin (AM, 10 μg), piperacillin-tazobactam (TPZ, 40 μg), cefazolin (CZ, 30 μg), cefoxitin (FOX, 30 μg), ceftriaxone (CRO, 30 μg), cefotaxime (CTX, 30 μg), ceftazidime (CAZ, 30 μg), cefepime (FEB, 30 μg), ceftaroline (CPT, 30 μg), imipenem (IPM, 10 μg), meropenem (MEM, 10 μg), nalidixic acid (NA, 10 μg), tobramycin (TOB, 10 μg), amikacin (AK, 30 μg), trimethoprim-sulphamethoxazole (SXT, 25 μg), chloramphenicol (C, 30 μg), tetracycline (TE, 30 μg), aztreonam (ATM, 30 μg), tigecycline (TIG, 15 μg), fosfomycin (FF, 50 μg), colistin (CT, 25 μg), ciprofloxacin (CIP, 5 μg), levofloxacin (LEV, 5 μg), and gentamicin (CN, 10 μg).

The multiple antibiotic resistance (MAR) index was determined for each isolate as previously described (Tambekar et al., 2006). The resistance phenotype of *Enterobacterales* isolates was defined as MDR, extensively drug-resistant (XDR), or pandrug-resistant (PDR) according to Magiorakos et al. (2012).

#### 2.4.2 Minimal inhibitory concentrations (MICs)

The minimum inhibitory concentrations (MICs) of IPM and MEM (Sigma-Aldrich, Seelze, Germany) for the isolates were determined using the broth microdilution method following CLSI guidelines and interpretative criteria (CLSI, 2022). *E. coli* ATCC25922 and *K. pneumoniae* ATCCBAA-1705 were included in the test as quality control strains. Isolates with MIC ≤1 µg/mL were interpreted as susceptible, intermediate if MIC =2 µg/mL, and resistant if MIC ≥4 µg/mL. Moreover, the MICs of CT and TIG over a concentration range of 0.125 to 64 μg/mL were determined following CLSI recommendations (CLSI, 2022). For CT, isolates were classified as susceptible if MIC was ≤2 µg/mL and resistant if MIC was >2 µg/mL according to CLSI/EUCAST Joint Working Group clinical breakpoints (EUCAST, 2016). However, isolates were classified as TIG susceptible if MIC was ≤2, intermediate if MIC was 4, and TIG resistant if MIC was ≥8 μg/mL, following the FDA susceptibility test interpretive criteria (FDA, 2023). The MIC_50_ (the concentration of the antibacterial agents that inhibited the growth of 50% of tested isolates) and the MIC_90_ (the concentration of the antibacterial agents that inhibited 90% of tested isolates) were then calculated using the ordered array method (Hamilton-Miller, 1991).

### 2.5 Phenotype detection of carbapenemase activity

Phenotypic testing for carbapenemase production was performed on all isolates that showed resistance or intermediate resistance to carbapenems using the Carba NP test (CNPt-direct), following the protocol recommended by Pasteran et al. (2015). Briefly, a bacterial colony from an overnight culture on Mueller-Hinton agar (MHA) was suspended in a 1.5-mL Eppendorf tube containing 100 μL of 0.1% (vol/vol) Triton X-100 (Sigma-Aldrich, Seelze, Germany) and mixed using vortex for 5-10 s. This lysate was then mixed with 100 μL of a phenol red solution in the reaction tube (consisting of 0.05% phenol red with 0.1 mmol/liter ZnSo_4_ previously adjusted to pH 7.8 and 6 mg/mL IPM) and a control tube (a phenol red solution without antibiotics). The tubes were mixed for an additional 5-10 s and incubated at 35°C. Isolates were interpreted as resistant if the color of the IPM-containing tube changed to orange-yellow.

### 2.6 Detection of carbapenem-resistance genes

#### 2.6.1 Molecular methods

All isolates phenotypically identified as resistant or intermediate resistant to carbapenems, according to the results of the IPM and MEM disc diffusion tests, were further confirmed by PCR assays for genes coding for carbapenem-hydrolyzing enzymes, including *bla*_KPC_, *bla*_IMP_, *bla*_NDM_, *bla*_VIM_, *bla*_OXA−48_, and *bla*_OXA-181_. The primer sets used in the PCR assays are listed in **Supplementary Table S2**.

DNA extraction was performed using the QIAamp DNA Mini Kit cat. no. 51304 (Qiagen, GmbH, Germany) following the manufacturer’s instructions. The concentrations of the extracted DNA were measured using NanoDrop™ 2000/2000c spectrophotometers (Thermo Fisher Scientific, Waltham, MA, USA).

PCR assays were performed using a T3 thermal cycler (Thermo Scientific, Waltham, MA, USA) in a 25-µL reaction mixture that contained 12.5 µL of EmeraldAmp Max PCR Master Mix (Takara, Shigino-higashi, Joto-ku, Osaka, Japan), 1 µL of each primer (20 pmol; Biobasic, Canada), 4.5 µL of nuclease-free water, and 6 µL of DNA template. Each run included a positive and negative control. PCR products were electrophoresed on a 1.5% agarose gel (Applichem GmbH, Darmstadt, Germany), and the gel was photographed using the Alpha Innotech gel documentation system (Biometra GmbH, Göttingen, Germany). PCR amplicons of 12 representative isolates (**Supplementary Table S3**) were selected for DNA sequencing based on geographical location, animal species, and breed. Amplicons were purified using a QIAquick PCR Purification Kit (QIAGEN, Crawley, UK) and sequenced using a Bigdye Terminator V3.1 cycle sequencing kit (Perkin-Elmer, Inc. Waltham, MA, USA) in an Applied Biosystems 3130 Genetic Analyzer (Hitachi, Tokyo, Japan). Nucleotide and deduced amino acid sequences were compared with those available in the National Center for Biotechnology Information using BLAST program (www.ncbi.nlm.nih.gov/BLAST/). The nucleotide sequences of KPC-2, IMP-1, VIM-1, NDM-1, NDM-5, OXA−48, and OXA-181 detected in this study for *K. pneumoniae* and *E. coli* isolates were deposited in GenBank database under accession numbers PP175341, PP175342, PP175345, PP158752, PP158751, PP175344, PP158750, PP175343, PP175346, PP158753, PP175347, and PP158749, respectively.

#### 2.6.2 NG-Test CARBA 5 immunochromatographic assay

Representative confirmed CPE isolates with different carbapenemase gene profiles (n = 16) and four PCR-negative isolates were subjected to further evaluation by NG-Test CARBA 5 kits (Changsha Zhong Sheng Zhong Jie Biotechnology Co.) following the instructions in the manufacturer’s manual. Briefly, a single colony of fresh isolate that had been cultured overnight on MHA was added to 150 µL of extraction buffer, vortexed for 5 s, and left at 25°C for 10 min. Subsequently, 100 µL of the prepared mixture was transferred to a sample well on a test card using a disposable pipette. After 15 min, the results were observed and interpreted. A positive result was indicated by the presence of a line in the control region (C) together with one or more lines in test regions K, O, V, I, and N. Each of these lines corresponded to the presence of KPC, OXA-48, VIM, IMP, or NDM-type carbapenemases, respectively. If the control line (C) did not appear, the reagent was deemed out of control, rendering the test invalid.

### 2.7 Data analysis

Descriptive statistics and data visualization were performed using R software (R Core Team, 2022; version 4.2.0). A heatmap of antimicrobial resistance patterns and resistance genes was created for the isolates using the “Complex heatmap” package (Gu et al., 2016). Chi-square and Fisher’s exact tests were used to assess the difference in the overall resistance proportion of the isolates recovered from the animals for each antimicrobial. Furthermore, the Wilcoxon-Mann-Whitney test was used to evaluate the differences in the MAR index of the isolates between dogs and cats; between healthy and diseased animals; and between confirmed and non-confirmed CRE isolates. The diagnostic characteristics, including sensitivity (Se), specificity (Sp), and overall accuracy, of the CNP-direct test and NG-Test CARBA 5 were calculated by comparing the results with those of other molecular methods. Furthermore, the Youden index (*J* = maximum [sensitivity + specificity-1]) and Cohen’s kappa statistic (*κ*) were determined to evaluate the applicability of the screening methods for detection of CRE.

## 3 Results

### 3.1 Detection of CRE

Out of 110 rectal swabs, 109 (99.1%) isolates were recovered from MEM-supplemented MacConkey agar, including 93 (84.6%) *K. pneumoniae*, 13 (11.8%) *E. coli*, and 3 (2.7%) *K. oxytoca* (**Figure 1**). Of these 109 isolates, 66 (60.6%) were confirmed as CRE by disc diffusion, broth microdilution, CNPt-direct, and PCR assays targeting carbapenemase genes including 56 (84.8%) *K. pneumoniae*, 7 (10.6%) *E. coli*, and 3 (4.6%) *K. oxytoca*.

Of the 66 CRE isolates, 43 (65.2%) were from dogs, and 23 (34.8%) from cats (**Table 1**). Additionally, CRE carriage was detected in 70% of healthy animals (including 27 dogs and 8 cats) and 52.5% of diseased animals (including 16 dogs and 15 cats). Notably, 57 (86.4%) of the CRE isolates were recovered from animals that had not received antimicrobials within the four weeks before sampling (**Table 1**).

**Table 1:** Number and percentage of carbapenem-resistant *Enterobacterales* (CRE) isolated from pets.

Parameters	Categories	N	No (%) of CRE isolates
Species			
	Dogs	62	43 (69.4)
	Cats	47	23 (48.9)
Health status			
	Healthy	50	35 (70.0)
	Diseased	59	31 (52.5)
Stray			
	Yes (Stray)	6	4 (66.7)
	No (domestic)	103	62 (60.2)
Locality			
	Cairo	59	46 (78.0)
	Dakahelia	40	18 (45.0)
	Sharkia	10	2 (20.0)
Gender			
	Male	47	30 (63.8)
	Female	50	33 (66.0)
	Unknown	12	3 (25.0)
Age			
	<1 year	65	42 (64.6)
	≥1 year	44	24 (54.6)
Antibiotic use within 4 weeks			
	Yes	24	9 (37.5)
	No	85	57 (67.1)
**Total**		**109**	**66 (60.6)**

### 3.2 Comparison of MAR index

**Figure 2:**
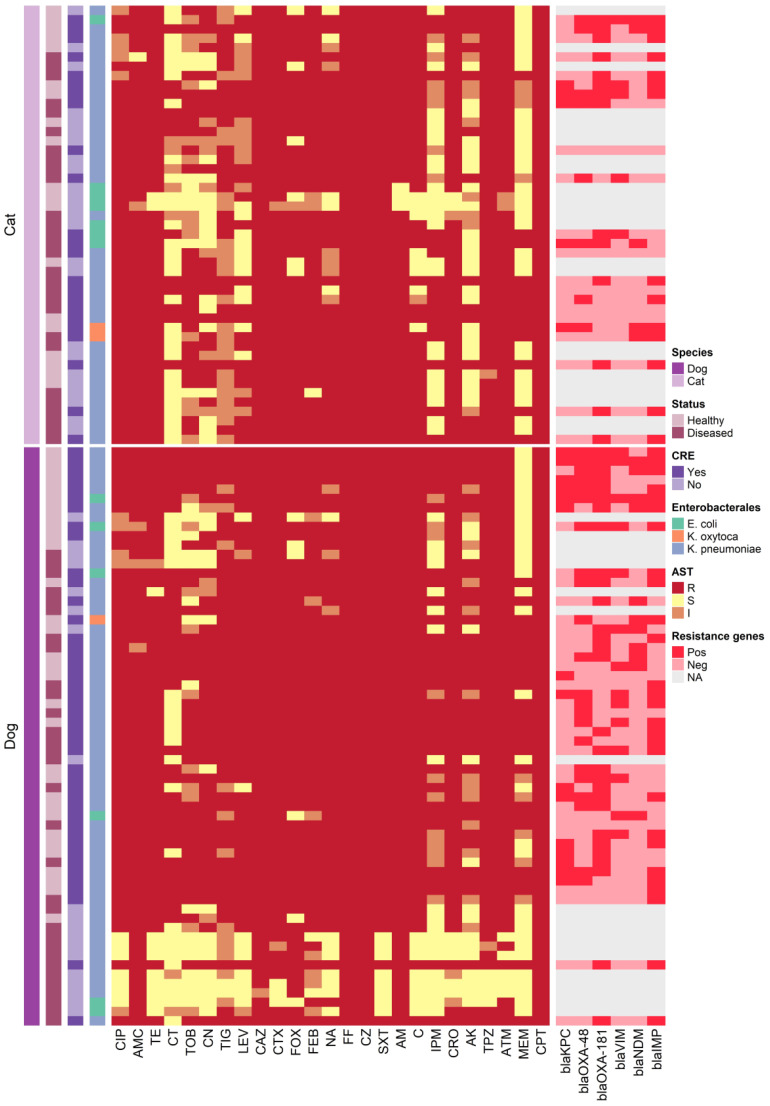
Heatmap representation of the isolates sources, antimicrobial resistance patterns and resistance genes.

**Figure 3:**
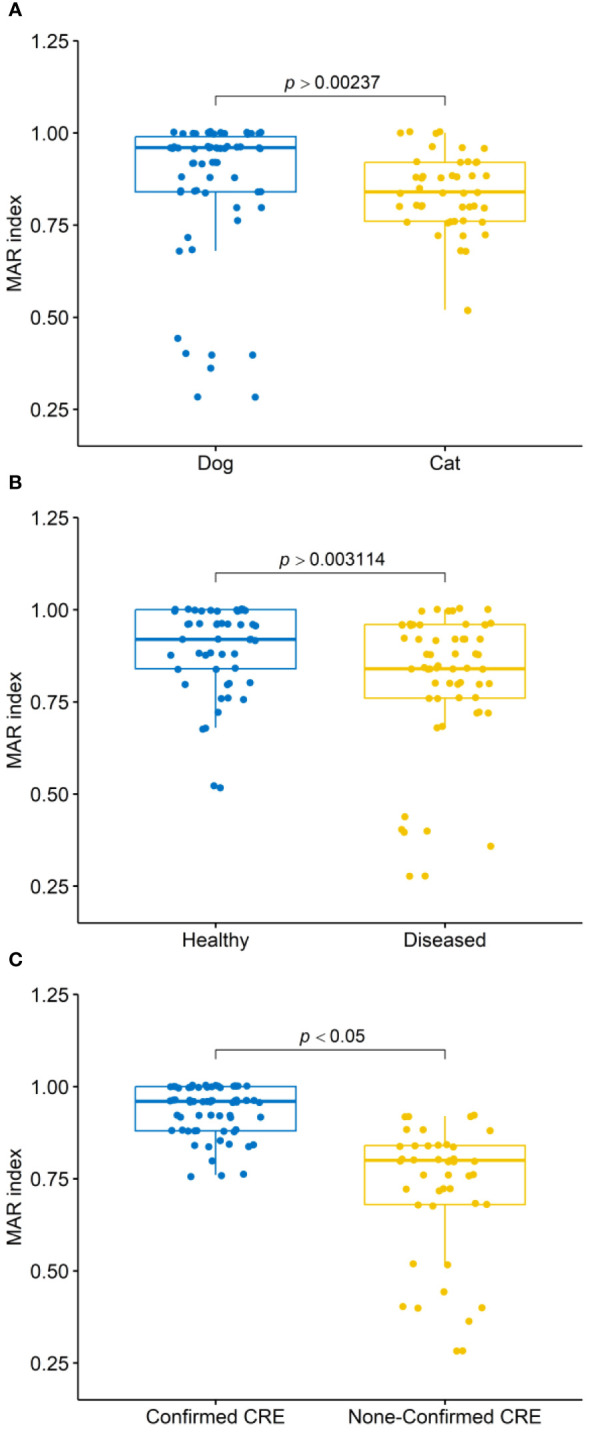
Box plots illustrate the multiple antibiotic resistance (MAR) index distributions of isolates: **(A)** from dogs and cats, **(B)** from healthy and diseased dogs and cats, and **(C)** confirmed as carbapenem-resistant *Enterobacterales* (CRE) and non-confirmed CRE. P-values obtained from Mann-Whitney U tests.

All 109 *Enterobacterales* isolates exhibited resistance to multiple tested antimicrobial agents (**Table 2** and **Figure 2**). Notably, 100% of the isolates were resistant to CPT, CAZ, CZ, and FF. High resistance proportions were observed to AMC (99.1%), TPZ (96.3%), CRO, and CIP (93.6%), AM, FEB, and CTX (97.2%), ATM (95.4%), and SXT (92.7%). The overall mean of MAR indices for CPE isolates was 0.85, ranging from 0.28 to 1.00. In dogs, the average MAR index of isolates was 0.86, significantly higher (*P >*0.002) than the 0.83 observed in cats (**Figure 3A**). Moreover, isolates from healthy dogs and cats had a significantly higher (*P >*0.003) average MAR index of 0.89 compared to 0.81 in diseased pets (**Figure 3B**). Significant differences were also found in the MAR index between CRE confirmed and non-confirmed isolates (**Figure 3C**). Of the CRE isolates, 52 (47.7%) were classified as MDR, 37 (33.9%) as XDR, and 20 (18.4%) as PDR, with MAR indices ranging from 0.28 to 0.88 for MDR and from 0.88 to 0.96 for XDR.

**Table 2:** Resistant proportions of 109 *Enterobacterales* isolates from dogs and cats.

Anti.^1^	No. (%) of all pets (n = 109)			No. (%) of dogs (n = 62)			No. (%) of cats (n = 47)			χ2 (*P*-value)
	*K. pneumoniae* (n = 93)	*E. coli* (n = 13)	*K. oxytoca* (n = 3)	*K. pneumoniae* (n = 55)	*E. coli* (n = 6)	*K. oxytoca* (n = 1)	*K. pneumoniae* (n = 38)	*E. coli* (n = 7)	*K. oxytoca* (n = 2)	
**CIP**	87 (93.6)	12 (92.3)	3 (100.0)	49 (89.1)	5 (83.3)	1 (100.0)	38 (100.0)	7 (100.0)	2 (100.0)	0.019
**AMC**	93 (100.0)	13 (100.0)	3 (100.0)	55 (100.0)	6 (100.0)	1 (100.0)	38 (100.0)	6 (85.7)	2 (100.0)	–
**TE**	86 (92.5)	9 (69.2)	3 (100.0)	48 (87.5)	4 (66.7)	1 (100.0)	38 (100.0)	5 (71.4)	2 (100.0)	0.110
**CT**	42 (45.2)	5 (38.5)	1 (33.3)	28 (50.9)	3 (50.0)	1 (100.0)	14 (36.8)	2 (28.6)	0 (0.0)	0.067
**TOB**	75 (80.7)	8 (61.5)	2 (66.7)	41 (74.6)	5 (83.3)	0 (0.0)	34 (89.5)	3 (42.9)	2 (100.0)	0.273
**CN**	70 (75.7)	5 (38.5)	1 (33.3)	44 (80.0)	3 (50.0)	0 (0.0)	26 (68.4)	2 (28.6)	1 (50.0)	0.112
**TIG**	64 (68.8)	6 (46.2)	1 (33.3)	43 (78.2)	4 (66.7)	1 (100.0)	21 (55.3)	2 (28.6)	0 (0.0)	0.002
**LEV**	76 (81.7)	9 (69.2)	2 (66.7)	47 (85.5)	5 (83.3)	1 (100.0)	29 (76.3)	4 (57.1)	1 (50.0)	0.090
**CAZ**	93 (100.0)	13 (100.0)	3 (100.0)	55 (100.0)	6 (100.0)	1 (100.0)	38 (100.0)	7 (100.0)	2 (100.0)	–
**CTX**	91 (97.9)	12 (92.3)	3 (100.0)	53 (96.4)	5 (83.3)	1 (100.0)	38 (100.0)	7 (100.0)	2 (100.0)	0.257
**FOX**	84 (90.3)	10 (76.9)	3 (100.0)	51 (92.7)	4 (66.7)	1 (100.0)	33 (86.8)	6 (85.7)	2 (100.0)	0.610
**FEB**	91 (97.9)	13 (100.0)	3 (100.0)	53 (96.4)	6 (100.0)	1 (100.0)	38 (100.0)	7 (100.0)	2 (100.0)	0.505
**NA**	84 (90.3)	10 (76.9)	3 (100.0)	48 (87.3)	5 (83.3)	1 (100.0)	36 (94.7)	5 (71.4)	2 (100.0)	0.549
**FF**	93 (100.0)	13 (100.0)	3 (100.0)	55 (100.0)	6 (100.0)	1 (100.0)	38 (100.0)	7 (100.0)	2 (100.0)	–
**CZ**	93 (100.0)	13 (100.0)	3 (100.0)	55 (100.0)	6 (100.0)	1 (100.0)	38 (100.0)	7 (100.0)	2 (100.0)	–
**SXT**	87 (93.6)	11 (84.6)	3 (100.0)	49 (89.1)	4 (66.7)	1 (100.0)	38 (100.0)	7 (100.0)	2 (100.0)	0.010
**AM**	93 (100.0)	10 (76.9)	3 (100.0)	55 (100.0)	6 (100.0)	1 (100.0)	38 (100.0)	4 (57.1)	2 (100.0)	0.077
**C**	82 (88.2)	9 (69.2)	2 (66.7)	49 (89.1)	4 (66.7)	1 (100.0)	33 (86.8)	5 (71.4)	1 (50.0)	0.547
**IPM**	55 (59.1)	7 (53.9)	3 (100.0)	37 (67.3)	4 (66.7)	1 (100.0)	18 (47.4)	3 (42.9)	2 (100.0)	0.047
**CRO**	88 (94.6)	11 (84.6)	3 (100.0)	50 (90.9)	6 (100.0)	1 (100.0)	38 (100.0)	5 (71.4)	2 (100.0)	0.696
**AK**	62 (66.7)	6 (46.2)	1 (33.3)	42 (76.4)	3 (50.0)	1 (100.0)	20 (52.6)	3 (42.9)	0 (0.0)	0.007
**TPZ**	90 (96.8)	12 (92.3)	3 (100.0)	52 (94.6)	5 (83.3)	1 (100.0)	38 (100.0)	7 (100.0)	2 (100.0)	0.132
**ATM**	88 (94.6)	13 (100.0)	3 (100.0)	50 (90.9)	6 (100.0)	1 (100.0)	38 (100.0)	7 (100.0)	2 (100.0)	0.069
**MEM**	41 (44.1)	3 (23.1)	3 (100.0)	28 (50.9)	1 (16.7)	1 (100.0)	13 (34.2)	2 (28.6)	2 (100.0)	0.202
**CPT**	93 (100.0)	13 (100.0)	3 (100.0)	55 (100.0)	6 (100.0)	1 (100.0)	38 (100.0)	7 (100.0)	2 (100.0)	–

^1^Anti., Antimicrobial agent; AMC, amoxicillin-clavulanic acid; AM, ampicillin; TPZ, piperacillin-tazobactam; CZ, cefazolin; FOX, cefoxitin; CRO, ceftriaxone; CTX, cefotaxime; CAZ, ceftazidime; FEB, cefepime; CPT, ceftaroline; IPM, imipenem; MEM, meropenem; NA, nalidixic acid; TOB, tobramycin; AK, amikacin; SXT, trimethoprim-sulphamethoxazole; C, chloramphenicol; TE, tetracycline; ATM, aztronam; TIG, tigecycline; FF, fosfomycin; CT, colistin; CIP, ciprofloxacin; LEV, levofloxacin; CN, gentamicin.

#### 3.2.1 MICs and CNPt-direct results of CRE isolates

The MICs of IPM ranged from 2 to 16 µg/mL for *Klebsiella* spp. and from 4 to 16 µg/mL for *E. coli*. while the MEM MICs ranged from 1 to 128 µg/mL for *Klebsiella* spp. and from 1 to 4 µg/mL for *E. coli* isolates (**Table 3**). The MIC_50_ for IPM was 8 µg/mL for *Klebsiella* spp. and 2 µg/mL for *E. coli*, while the MIC_50_ for MEM was 2 µg/mL for both *Klebsiella* spp. and *E. coli*. The proportions of IPM and MEM resistance were higher among *Klebsiella* spp., compared to *E. coli* isolates. The *Klebsiella* spp. had approximately 1.3-fold higher for MIC_50_ and MIC_90_ for carbapenems (**Table 3**).

**Table 3:** Minimum inhibitory concentration (MIC) values of imipenem and meropenem for carbapenem-resistant *Enterobacterales* isolates.

Isolates	Antibiotic	R (%)^1^	95% CI	MIC for resistant isolates (µg/mL)^2^			
				MIC_50_	MIC_90_	GM	Range
***Klebsiella* spp. (n = 96)**	Imipenem	58 (60.4)	49.9 – 70.3	8	16	6.6	2 - 16
	Meropenem	44 (45.8)	35.6 – 56.3	2	128	4.9	1 - 128
***E. coli* (n = 13)**	Imipenem	7 (53.8)	25.1 - 80.8	8	16	6.4	4 - 16
	Meropenem	3 (23.1)	5.0 - 53.8	2	4	6.5	1 - 4
**Total (n = 109)**	Imipenem	65 (59.6)	49.8 – 68.9	8	16	6.6	2 - 16
	Meropenem	47 (43.1)	33.7 – 52.9	2	128	4.9	1 - 128

^1^R: number of resistant isolates.

^2^MIC_50_, MIC values that inhibited 50% of the isolates; MIC_90_, MIC values that inhibited 90% of the isolates; GM, geometric mean.

Of the 66 CRE isolates, 56 (84.8%) did not show susceptibility to MEM with MIC values ranging from 2 to 128 µg/mL. Interestingly, 16 isolates (28.6%), initially classified as MEM-susceptible by disc diffusion testing, demonstrated non-susceptibility when assessed by MIC (**Table 4**). Conversely, 7 of the 66 CRE isolates (10.6%) showed susceptibility to MEM with an MIC of 1 µg/mL despite being initially identified as non-susceptible by disc diffusion testing.

**Table 4:** Distribution of carbapenem resistance genes in 66 carbapenem-resistant *Enterobacteriales* isolates.

N	Resistance genes	MIC (µg/mL)^1^		Disc diffusion^2^		Carab NP^2^
		IPM	MEM	IPM	MEM	
*Escherichia coli* (n = 7)						
2	VIM+ IMP+OXA-48/OXA-181	4-16	2-4	1 (I) 1 (R)	2 (S)	1 (R) 1 (S)
1	KPC+VIM+NDM+IMP+OXA-48-like	8	2	1 (I)	1 (S)	1 (R)
1	VIM+NDM+IMP+OXA-48-like	8	2	1 (R)	1 (S)	1 (R)
1	VIM+NDM	4	1	1 (R)	1 (R)	1 (R)
1	VIM+OXA-181	8	2	1 (R)	1 (R)	1 (R)
1	KPC+NDM+OXA-48-like	8	2	1 (R)	1 (R)	1 (S)
*Klebsiella oxytoca* (n = 3)						
1	NDM+IMP+OXA-48	4	2	1 (R)	1 (R)	1 (R)
1	KPC+NDM+IMP+OXA-48	8	2	1 (R)	1 (R)	1 (R)
1	NDM+IMP	8	2	1 (R)	1 (R)	1 (R)
*Klebsiella pneumoniae* (n = 56)						
11	OXA-181+IMP	2-8	1-128	2 (I) 9 (R)	2 (S) 9 (R)	5 (R) 6 (S)
3	KPC+OXA-181	4-8	1-128	2 (I) 1 (R)	2 (S) 1 (I)	3 (R)
3	KPC+OXA-48-like+VIM+NDM+IMP	4-8	1-2	1 (I) 2 (R)	1 (S) 1 (I) 1(R)	2 (R) 1 (S)
3	IMP	8-16	2-4	1 (I) 2 (R)	1 (I) 2 (R)	3 (R)
2	OXA-48+VIM+IMP	8-16	4-128	2 (R)	2 (R)	2 (R)
2	KPC+OXA-48/OXA-181	4-16	2-4	1 (I) 1 (R)	1 (S) 1 (I)	1 (R) 1 (S)
2	VIM+NDM	8-16	2	2 (R)	1 (S) 1 (R)	2 (R)
2	OXA-181+NDM	4	128	2 (R)	2 (R)	2 (S)
2	OXA-48-like+NDM+IMP	8-16	2-4	2 (R)	2 (S)	1 (R) 1 (S)
2	OXA-181+VIM+IMP	4	1-128	1 (I) 1 (R)	1 (I) 1 (R)	2 (R)
2	OXA-48+IMP	4-8	2-128	2 (R)	2 (R)	2 (R)
2	OXA-48-like	4	2-128	2 (R)	2 (R)	2 (R)
2	KPC+OXA-48-like+VIM+IMP	4	1-2	1 (I) 1 (R)	1 (S) 1 (I)	1 (R) 1 (S)
1	OXA-48-like+IMP	8	128	1 (R)	1 (R)	1 (S)
1	OXA-181, VIM, NDM	4	128	1 (S)	1 (R)	1 (S)
1	KPC+OXA-181+NDM+IMP	8	2	1 (R)	1 (S)	1 (S)
1	KPC+OXA-181+IMP	8	2	1 (I)	1 (S)	1 (R)
1	KPC+OXA-181+VIM+IMP	8	128	1 (I)	1 (I)	1 (R)
1	KPC+OXA-48-like+VIM+NDM+IMP	8	2	1 (R)	1 (S)	1 (S)
1	KPC+OXA-181+VIM+IMP	8	128	1 (I)	1 (I)	1 (R)
1	KPC+OXA-48+IMP	8	4	1 (R)	1 (R)	1 (R)
1	OXA-48-like+NDM	16	2	1 (R)	1 (R)	1 (R)
1	OXA-48-like+VIM	8	128	1 (I)	1 (I)	1 (R)
1	OXA-48+VIM	2	1	1 (I)	1 (I)	1 (S)
1	KPC	16	2	1 (R)	1 (R)	1 (S)
1	OXA-48	8	128	1 (R)	1 (R)	1 (R)
1	NDM	8	2	1 (R)	1 (R)	1 (R)
4	–	2-8	1-2	1 (I) 3 (R)	1 (S) 3 (R)	2 (R) 2 (S)

^1^MIC, minimum inhibitory concentration; IPM, imipenem; MEM, meropenem.

^2^S, sensitive; I, intermediate; R, resistant.

#### 3.2.2 Antibiogram signature of CRE

**Table 5** displays the antimicrobial resistance profiles of the 66 CRE isolates, revealing 28 distinct antibiogram patterns, including PDR (n = 20), XDR (n = 30), and MDR (n = 16) phenotypes. The MICs of CT for these CRE isolates varied from 0.5 µg/mL to 32 µg/mL, while TIG MICs ranged from 1 µg/mL to 64 µg/mL. Notably, among the MDR and XDR CRE isolates (comprising 14 *K. pneumoniae*, one *E. coli*, and one *K. oxytoca*) recovered from cases involving diarrhea and/or vomiting, there was susceptibility to at least one antimicrobial drug, including CT, TIG, LEV, and AK. Furthermore, among the 35 CRE isolates (comprising 29 *K. pneumoniae*, four *E. coli*, and two *K. oxytoca*) from healthy pets, the MAR index ranged from 0.76 to 1. This group included seven MDR and 14 XDR isolates that exhibited susceptibility to at least one non-carbapenem antibiotic, such as CT, TIG, and CN.

**Table 5:** Antibiogram signature of carbapenem-resistant *Enterobacteriales* isolates recovered from dogs and cats.

Patterns	No. of isolates	Bacterial spp.	Source	Resistance pattern^1^	MAR Index^2^	Resistance phenotype	MIC (µg/mL)				Carbapenemase genes
							IPM	MEM	CT	TIG	
1	20	*K. pneumoniae*	Dog	CIP, AMC, TE, CT, TOB, CN, TIG, LEV, CAZ, CTX, FOX, FEB, NA, FF, CZ, SXT, AM, C, IPM, CRO, AK, TPZ, ATM, MEM, CPT	1	PDR	8	128	16	8	*bla*_OXA-48_, *bla*_OXA-181_, *bla*_IMP_
			Dog				8	128	32	64	*bla*_OXA-181_, *bla*_IMP_
			Dog				4	128	32	16	*bla*_OXA-181_, *bla*_NDM_
			Dog				16	2	32	16	*bla*_OXA-48,_ *bla_O_*_XA-181_, *bla*_NDM_
			Dog				16	2	16	32	*bla*_NDM_, *bla*_VIM_
			Dog				16	2	16	8	*bla*_KPC_
			Dog				4	128	16	32	*bla*_OXA-181_, *bla*_IMP_, *bla*_VIM_
			Dog				8	128	32	4	*bla*_OXA-48_, *bla*_OXA-181_, *bla*_VIM_
			Dog				4	1	32	4	*bla*_KPC,_ *bla*_OXA-48_, *bla*_OXA-81,_ *bla*_IMP_
			Cat				4	1	32	4	*bla*_KPC_, *bla*_OXA-48_, *bla*_OXA-81_, *bla*_VIM,_ *bla*_IMP_
			Dog				4	2	16	4	*bla*_OXA-48_, *bla*_OXA-181_
			Dog				4	1	16	32	Negative
			Dog				4	1	32	4	*bla*_OXA-181_, *bla*_VIM_
			Dog				8	1	16	32	*bla*_KPC,_ *bla*_OXA-48_, *bla*_OXA-181_, *bla*_VIM_, *bla*_IMP_
			Dog				8	4	32	16	*bla*_KPC,_ *bla*_OXA-48_, *bla*_IMP_
			Dog				8	4	16	64	*bla*_IMP_
			Dog				8	4	32	4	*bla*_IMP_
			Cat				8	2	32	32	*bla*_OXA-181_, *bla*_IMP_
			Cat				8	2	32	64	Negative
			Cat				8	2	16	32	*bla*_OXA-181_, *bla*_IMP_
2	7	*K. pneumoniae*	Dog	CIP, AMC, TE, CT, TOB, CN, TIG, LEV, CAZ, CTX, FOX, FEB, NA, FF, CZ, SXT, AM, C, IPM, CRO, AK, TPZ, ATM, CPT	0.96	XDR	4	2	8	16	*bla*_KPC_, *bla*_OXA-48_, *bla*_OXA-181_, *bla*_IMP_, *bla*_VIM_
			Dog				8	2	16	16	*bla*_KPC,_ *bla*_OXA-48_, *bla*_OXA-181,_ *bla*_VIM,_ *bla*_IMP,_ *bla*_NDM_
			Dog				8	2	8	8	*bla*_OXA-48_, *bla*_OXA-181_, *bla*_IMP_
			Dog				16	4	16	8	*bla*_KPC_, *bla*_OXA-48_, *bla*_OXA-181_
			Cat				16	4	16	16	*bla*_OXA-48_, *bla*_OXA-181_, *bla*_IMP_, *bla*_NDM_
		*E. coli*	Dog				16	4	32	64	*bla*_OXA-48_, *bla*_OXA-181_, *bla*_VIM,_ *bla*_IMP_
		*K. pneumoniae*	Dog				8	2	32	4	*bla*_KPC_, *bla*_OXA-181_, *bla*_IMP_
3	3	*K. pneumoniae*	Dog	CIP, AMC, TE, TOB, CN, LEV, CAZ, CTX, FOX, FEB, NA, FF, CZ, SXT, AM, C, IPM, CRO, AK, TPZ, ATM, CPT	0.88	MDR	8	2	1	2	*bla*_KPC_, *bla*_OXA-48_, *bla*_OXA-181_, *bla*_IMP_
		*E. coli*	Cat				8	2	1	1	*bla*_OXA-48_, *bla*_OXA-181_, *bla*_VIM_, *bla*_IMP_, *bla*_NDM_
		*K. pneumoniae*	Dog				4	1	2	2	*bla*_KPC_, *bla*_OXA-181_
4	2	*E. coli*	Dog	CIP, AMC, TE, CT, TOB, CN, TIG, LEV, CAZ, CTX, FOX, FEB, NA, FF, CZ, SXT, AM, C, IPM, CRO, TPZ, ATM, CPT	0.92	XDR	8	2	16	4	*bla*_KPC_, *bla*_OXA-48,_ *bla*_OXA-181_, *bla*_VIM_, *bla*_IMP_, *bla*_NDM_
			Cat				2	1	8	4	Negative
5	1	*K. pneumoniae*	Cat	CIP, AMC, TE, CT, CN, LEV, CAZ, CTX, FOX, FEB, NA, FF, CZ, SXT, AM, C, IPM, CRO, AK, TPZ, ATM, CPT	0.88	MDR	8	2	16	2	*bla*_KPC_, *bla*_OXA-181_, *bla*_IMP_, *bla*_NDM_
6	1	*E. coli*	Dog	CIP, AMC, TE, TOB, TIG, LEV, CAZ, CTX, FOX, FEB, NA, FF, CZ, SXT, AM, C, IPM, CRO, TPZ, ATM, CPT	0.84	MDR	4	2	2	16	*bla*_OXA-181_, *bla*_IMP_
7	1	*K. pneumoniae*	Cat	CIP, AMC, TE, TOB, CN, TIG, CAZ, CTX, FOX, FEB, NA, FF, CZ, SXT, AM, C, IPM, CRO, AK, TPZ, ATM, CPT	0.84	MDR	2	2	2	4	*bla*_OXA-181,_ *bla*_IMP_
8	1	*K. pneumoniae*	Cat	CIP, AMC, TE, TIG, LEV, CAZ, CTX, FOX, FEB, NA, FF, CZ, SXT, AM, C, IPM, CRO, AK, TPZ, ATM, CPT	0.87	MDR	4	2	2	4	*bla*_OXA-181_, *bla*_IMP_
9	8	*K. pneumoniae*	Dog	CIP, AMC, TE, TOB, CN, TIG, LEV, CAZ, CTX, FOX, FEB, NA, FF, CZ, SXT, AM, C, IPM, CRO, AK, TPZ, ATM, MEM, CPT	0.96	XDR	16	2	1	32	*bla*_IMP_
							16	4	1	32	*bla*_OXA-48_, *bla*_IMP_, *bla*_VIM_
							8	128	2	64	*bla*_OXA-48_
							8	128	1	16	*bla*_OXA-48_ *bla*_IMP,_ *bla*_VIM_
							4	128	2	32	*bla*_OXA-181_, *bla*_IMP_
							4	128	2	16	*bla*_VIM,_ *bla*_NDM_
							8	4	1	8	*bla*_OXA-181_, *bla*_IMP_
							8	4	2	16	*bla*_OXA-181_, *bla*_IMP_
10	2	*K. pneumoniae*	Cat	CIP, AMC, TE, TOB, CN, LEV, CAZ, CTX, FOX, FEB, NA, FF, CZ, SXT, AM, C, IPM, CRO, AK, TPZ, ATM, MEM, CPT	0.92	XDR	8	4	1	2	*bla*_OXA-181_, *bla*_IMP_
							4	2	1	2	*bla*_OXA-181_, *bla*_IMP_
11	1	*K. pneumoniae*	Dog	CIP, AMC, TE, CT, CN, TIG, LEV, CAZ, CTX, FOX, FEB, NA, FF, CZ, SXT, AM, C, IPM, CRO, AK, TPZ, ATM, MEM, CPT	0.96	XDR	4	128	32	16	*bla*_OXA-181_, *bla*_NDM_
12	1	*K. oxytoca*	Dog	CIP, AMC, TE, CT, TIG, LEV, CAZ, CTX, FOX, FEB, NA, FF, CZ, SXT, AM, C, IPM, CRO, AK, TPZ, ATM, MEM, CPT	0.92	XDR	4	2	16	4	*bla*_OXA-181_, *bla*_IMP_, *bla*_NDM_
13	1	*K. pneumoniae*	Dog	CIP, AMC, TE, CT, TOB, CN, TIG, LEV, CAZ, CTX, FOX, FEB, NA, FF, CZ, SXT, AM, C, CRO, TPZ, ATM, MEM, CPT	0.92	XDR	4	128	32	4	*bla*_OXA-181_, *bla*_NDM_, *bla*_VIM_
14	1	*K. pneumoniae*	Dog	CIP, AMC, TE, TOB, CN, TIG, LEV, CAZ, CTX, FOX, FEB, NA, FF, CZ, SXT, AM, C, IPM, CRO, AK, TPZ, ATM, CPT	0.92	XDR	16	4	1	16	*bla*_KPC,_ *bla*_OXA-48_, *bla*_IMP_, *bla*_VIM_
15	3	*K. pneumoniae*	Dog	CIP, AMC, TE, CT, TOB, TIG, LEV, CAZ, CTX, FOX, FEB, NA, FF, CZ, SXT, AM, C, IPM, CRO, AK, TPZ, ATM, MEM, CPT	0.96	XDR	4	128	32	4	*bla*_OXA-48_, *bla*_OXA-181_
			Cat				8	128	32	4	*bla*_OXA-181_, *bla*_IMP_, *bla*_VIM_
			Cat				8	2	16	32	Negative
16	1	*E. coli*	Dog	CIP, AMC, TE, CT, TOB, CN, LEV, CAZ, CTX, FEB, NA, FF, CZ, SXT, AM, C, IPM, CRO, AK, TPZ, ATM, MEM, CPT	0.92	XDR	4	1	32	2	*bla*_VIM,_ *bla*_NDM_
17	1	*K. pneumoniae*	Dog	CIP, AMC, TE, CT, TOB, CN, TIG, LEV, CAZ, CTX, FOX, FEB, NA, FF, CZ, SXT, AM, C, IPM, CRO, TPZ, ATM, MEM, CPT	0.96	XDR	8	2	16	4	*bla*_KPC_, *bla*_OXA-181_
18	1	*K. pneumoniae*	Cat	CIP, AMC, TE, TOB, CN, TIG, LEV, CAZ, CTX, FOX, FEB, NA, FF, CZ, SXT, AM, C, IPM, CRO, TPZ, ATM, MEM, CPT	0.92	XDR	4	2	2	4	*bla*_KPC,_ *bla*_OXA-48_, *bla*_OXA-181_
19	1	*K. pneumoniae*	Cat	CIP, AMC, TE, TIG, LEV, CAZ, CTX, FOX, FEB, NA, FF, CZ, SXT, AM, C, IPM, CRO, AK, TPZ, ATM, MEM, CPT	0.88	MDR	2	1	1	8	*bla*_OXA-48_, *bla*_VIM_
20	1	*E. coli*	Cat	CIP, AMC, TE, CT, TOB, TIG, CAZ, CTX, FOX, FEB, NA, FF, CZ, SXT, AM, C, IPM, CRO, TPZ, ATM, MEM, CPT	0.85	XDR	8	2	32	4	*bla*_OXA-181_, *bla*_VIM_

^1^AMC, amoxicillin-clavulanic acid; AM, ampicillin; TPZ, piperacillin-tazobactam; CZ, cefazolin; FOX, cefoxitin; CRO, ceftriaxone; CTX, cefotaxime; CAZ, ceftazidime; FEB, cefepime; CPT, ceftaroline; IPM, imipenem; MEM, meropenem; NA, nalidixic acid; TOB, tobramycin; AK, amikacin; SXT, trimethoprim-sulphamethoxazole; C, chloramphenicol; TE, tetracycline; ATM, aztronam; TIG, tigecycline; FF, fosfomycin; CT, colistin; CIP, ciprofloxacin; LEV, levofloxacin; and CN, gentamicin.

^2^MAR index, multiple-antibiotic resistance index.

^3^MDR, multidrug-resistant; XDR, extensively drug-resistant; PDR, pandrug-resistant.

#### 3.2.3 Pandrug-resistant *K. pneumoniae*

Twenty *K. pneumoniae* isolates did not display susceptibility to all tested antimicrobials, encompassing aminoglycosides, penicillins, penicillins with *β*-lactamase inhibitors, cephalosporins, anti-MRSA cephalosporins, cephamycins, fluoroquinolones, folate pathway inhibitors, glycylcyclines, phenicols, carbapenems, monobactams, antipseudomonal penicillins with *β*-lactamase inhibitors, polymyxins, tetracyclines, and phosphonic acids (**Table 5**). The MICs of CT and TIG for these PDR CR *K. pneumoniae* isolates ranged from 16 µg/mL to 32 µg/mL and 4 µg/mL to 64 µg/mL, respectively. These PDR isolates were collected from 14 healthy pets (11 dogs and three cats) and six diseased pets, among which two had respiratory tract infections and four had diarrhea.

### 3.3 Carbapenemase encoding genes in CRE

Sixty-six CRE isolates, initially identified by disc diffusion, MIC, and CNPt-direct testing, were subjected to examination for carbapenemase-encoding genes. Among them, 62 isolates (93.9%) tested positive for at least two of the six carbapenemase-encoding genes assessed (**Table 4**). The most frequently encountered gene in CRE isolates was *bla*_OXA-181_, detected in 42 out of 66 isolates (63.6%), followed by *bla*_IMP_ which identified in 40 (60.6%) isolates, while *bla*_OXA−48-like_ was found in 29 (43.9%) isolates. Additionally, both *bla*_KPC_ and *bla*_VIM_ were present in 20 (30.3%) of the isolates, and *bla*_NDM_ was detected in 17 (25.8%) isolates. DNA sequencing and sequence analysis of the amplicons of carbapenemase-encoding genes revealed KPC-2, IMP-1, VIM-1, NDM-1, and NDM-5 genotypes.

Twenty representative isolates including 16 positives for carbapenemase-encoding genes (cover all tested genes; 8 KPC producing-isolates, 14 from OXA, 5 from NDM, 9 from IMP, and 5 from VIM producing-isolates) and four negative isolates were tested with NG-Test CARBA 5 (**Figure 1** and **Supplementary Table S4**). Eight isolates were confirmed to harbor the *bla*_KPC_ gene. Thirteen isolates were positive for OXA, except for one *K. pneumoniae* isolate (code no D8), that harbored *bla*_OXA-181_, was false-negative by NG-Test CARBA 5. Five isolates were considered to have a true-positive result for NDM and VIM. Moreover, seven isolates were true-positive for IMP. Two *K. pneumoniae* isolates were false-positive for VIM (code no D1 and D23); also, D1 isolate was false-positive for KPC. Two isolates were false-negative for IMP (code no D14 and D10; **Supplementary Table S4**).

### 3.4 Association between carbapenemase-encoding genes and MICs

*K. pneumoniae* and *E. coli* isolates harboring *bla*_KPC_ (n = 20) were non-susceptible to IPM (MIC_90_ 16 µg/mL) and 16/20 (80%) were non-susceptible to MEM (MIC_90_ 4 µg/mL), while those harboring *bla*_VIM_ (n = 19) were non-susceptible to IPM (MIC_90_ 16 µg/mL) and 15/19 (78.9%) non-susceptible to MEM (**Table 6**). Moreover, 100% of isolates positive for *bla*_NDM_ or *bla*_IMP_, *bla*_OXA−48,_ and *bla*_OXA-181_ were non-susceptible to IPM, but 93.8%, 87.8%, 86.7%, and 86% of these isolates were MEM non-susceptible, respectively.

**Table 6:** Relationship between carbapenem resistance genes and MICs of imipenem and meropenem against isolates.

Paramter^1^	MIC of IPM (µg/mL)						MIC of MEM (µg/mL)					
	KPC	VIM	NDM	IMP	OXA-48	OXA-181	KPC	VIM	NDM	IMP	OXA-48	OXA-181
**GM**	7.5	6.9	7.7	6.9	7.1	6.3	2.6	9.5	5.8	6.3	6.9	9.1
**MIC_50_**	8	8	8	8	8	8	2	2	2	2	2	2
**MIC_90_**	16	16	16	16	16	16	4	128	128	128	128	128
**MIC range**	4 - 16	2 - 16	4 - 16	2 - 16	2 - 16	2 - 16	1 - 128	1 - 128	1 - 128	1 - 128	1 - 128	1 - 128

^1^GM, geometric mean; MIC_50_, MIC values that inhibited 50% of the isolates; MIC_90_, MIC values that inhibited 90% of the isolates.

### 3.5 Diagnostic characteristics of CRE screening methods

The diagnostic characteristics of CNPt-direct and CARBA 5 assays compared to PCR (as the reference test) were evaluated for the detection of CRE isolates (**Table 7**). CNPt-direct assay showed lower Se, Sp, and accuracy than that of CARBA 5 assay. Furthermore, the CARBA 5 assay showed higher Se and Sp for the detection of CRE genes (NDM, OXA and KPC) than that for VIM and IMP genes.

**Table 7:** Diagnostic test characteristics of screening methods for detection of carbapenem-resistant *Enterobacterales*.

Test	Diagnostic characteristics^1^			*J^2^*	*K^3^*	PCR +/-	Test +/-
	Se (95% CI)	Sp (95% CI)	Accuracy				
**Carba NP**	67.7 (54.7 – 79.1)	50.0 (6.8 – 79.1)	66.7	0.18	0.06	62/4	44/22
CARBA 5							
**KPC**	100 (63.1 – 100)	91.7 (61.5 – 99.8)	95.0	0.92	0.90	8/12	9/11
**OXA**	92.9 (66.1 – 99.8)	100 (54.1 – 100)	95.0	0.93	0.89	14/6	13/7
**VIM**	83.3 (35.9 – 99.6)	85.7 (57.2 – 98.2)	85.0	69	0.66	6/14	7/13
**NDM**	100 (47.8 – 100)	100 (78.2 – 100)	100	100	1.0	5/15	5/15
**IMP**	77.8 (40.0 – 97.2)	100 (71.5 - 100)	90	77.8	0.79	9/11	7/13

^1^Se, sensitivity; Sp, specificity; PPV, positive predictive value; NPV, negative predictive value.

^2^J: Youden index.

^3^K: Cohen’s kappa.

## 4 Discussion

The emergence and global spread of CRE are of great concern, with reservoirs expanding not only within hospitals but also in the community and the environment (Nigg et al., 2019; Sankar et al., 2022; Silva et al., 2022; Bulens et al., 2023). The scientific community has paid attention to the occurrence of CRE in animals due to the severe impact of this phenomena (Wang et al., 2017; Pulss et al., 2018). To curb the spread of high-risk clones in humans, animals, and the environment, veterinary settings must implement an early detection, worldwide surveillance of CRE, as well as efficient infection prevention and control strategies (Nigg et al., 2019). Therefore, this study aims to assess the prevalence of CRE among healthy and diseased pets in Egypt, evaluated the performance of three screening methods (meropenem-supplemented MacConkey agar, CNPt-direct, and NG-Test CARBA 5) for CRE detection, and characterized the antimicrobial resistance of these isolates. Notably, this research not only strengthens the limited resistance data but also represents the first study to investigate the NG-Test CARBA 5 assay for rapid CRE detection in pets in Egypt.

A recent study highlights an emerging trend of MDR in 50% of isolates from companion animals, raising concerns about the potential for cases that may not respond to first-line antimicrobials in the near future (Martins et al., 2022). Similarly, our findings revealed a high rate of antimicrobial resistance (AMR) among *Enterobactereales* isolates recovered from companion animals in Egypt. The driving force for this situation is the misuse of antibiotics in the veterinary sector (Caneschi et al., 2023; W.H.O, 2023), where amoxicillin-clavulanic acid and 1^st^ generation cephalosporins are among the most frequently prescribed medications for dogs (Murphy et al., 2012). Furthermore, *β*-lactam antibiotics are frequently used to treat bacterial infections in pets (Rubin and Pitout, 2014). Although carbapenems are not licensed for use in veterinary medicine, CRE and CPE have been reported in animals (Anderson and Boerlin, 2020; Silva et al., 2022). The Centers for Disease Control and Prevention recommended rectal swabs screening to identify carriers of carbapenem-resistant GNB and implement appropriate infection control measures (CDC, 2009). Culture-based methods have been widely used for CRE screening in clinical laboratories (Ambretti et al., 2019). The use of enrichment broth containing carbapenem discs enhances CRE-carrier detection sensitivity in humans due to the instability of carbapenems in liquid solutions (Glaser et al., 2015; Darling et al., 2017). Similar benefits could also be expected in identifying carrier animals (Anderson and Boerlin, 2020).

In this study, 109 colonies recovered from MEM-supplemented MacConkey agar were further confirmed, of which 66 (60.6%) were identified as CRE and 43 as CRE-negative. A previous study reported that the CDC method led to a significant number of MEM-susceptible colonies on MacConkey plates, with only 26% (29/111) of the lactose-fermenting colonies being CREs (Glaser et al., 2015). Moreover, Darling et al. (2017) identified 290/483 (60%) CRE-negative false turbid TSB supplemented with ertapenem, and among these, 47 were falsely identified as CRE-negative when TSB was combined with ertapenem (ETP), fluconazole, and linezolid.

The use of the disc diffusion method for identifying CREs and CPEs in animals is supported by several studies (Lee and Chung, 2015; Haldorsen et al., 2018), which have revealed a strong correlation between disc diffusion and broth microdilution methods. In the present study, 28.6% of the CRE isolates, initially identified as MEM-susceptible through the disc diffusion test, were subsequently found to be non-susceptible based on the MIC results. Additionally, 10.6% of the CRE isolates, which harbor OXA-48 and/or OXA-181 and were initially identified as non-susceptible by the disc diffusion test (3 resistant and 4 intermediate), were found to be susceptible to MEM (MIC 1 µg/mL). Similarly, Sekar et al. (2019) observed that 24.2% of isolates susceptible to ETP based on MIC results were identified as non-susceptible by the disc diffusion test. This discrepancy can be explained by the fact that many OXA-48 producers exhibit low MICs for carbapenems (often susceptible to IPM, MEM, and intermediate or susceptible to ETP) (Tamma et al., 2016; Tamma and Simner, 2018). Consequently, the disk diffusion test is more sensitive than the ‘true’ MIC for detecting carbapenemases of the OXA-48 family (Sekar et al., 2019). Seven isolates were positive for metallo-β lactamases and oxacillinases, as revealed in **Table 5**, these were MEM sensitive (MIC 1 µg/mL) and IPM resistant (MIC 4-8 µg/mL**),** but one was intermediate to IPM (MIC 2 µg/mL). Similarly, Fattouh et al. (2016) reported that 19 *E. coli* and 4 *K. pneumoniae* isolates that were positive for all carbapenemases (*bla*_KPC_, *bla*_NDM_, *bla*_OXA-48_, *bla*_GES_, *bla*_VIM_, and *bla*_IMP_) had low MEM MIC (0.25-1 µg/mL). Additionally, *K. pneumoniae* producing KPC, NDM, and OXA-48 carbapenemases frequently exhibited moderate to high MICs (MIC > 2 µg/mL). Nordmann et al. (2011) reported that the resistance levels to metallo-β lactamases producers may vary (MEM MIC: 0.25 – >64) and low-level resistance and even susceptibility to carbapenems have been observed for producers of any type of carbapenemase. This could further promote CPE dissemination. Therefore, detecting the presence of carbapenemase genes is important even for those isolates deemed susceptible to carbapenems (Fattouh et al., 2016).

Although the molecular identification of carbapenemase-encoding genes is the reference method, the phenotypic detection of carbapenem resistance is a feasible alternative for routine diagnosis (Silva et al., 2022). Multiple methods, including inhibitor-based methods, inactivation of carbapenems, detection of carbapenem hydrolysis products, immunochromatographic assays, and Matrix-Assisted Laser Desorption/Ionization-Time of Flight Mass Spectrometry (MALDI-TOF), have been developed to confirm carbapenemase production and differentiate between CPEs and other types of CREs (Anderson and Boerlin, 2020).

In this study, CNPt-direct was used to detect carbapenem hydrolysis and was chosen due to its ease of use and direct utilization of colonies for rapid carbapenemase production detection (Pasteran et al., 2015). The CNPt-direct identified 70.97% (44/62) of the CPE isolates. Notably, false-negative results were observed for OXA producers (17 *K. pneumoniae* and 2 *E. coli*) and one KPC-producing *K. pneumoniae* isolate. Additionally, two false-positive results were obtained. These findings were consistent with several studies that have reported satisfactory performance of the CNPt-direct or Carba NP test. However, one of the main limitations of these assays is their relatively lower sensitivity for detecting OXA producers (Tijet et al., 2013; Osterblad et al., 2014; CLSI, 2015; Pasteran et al., 2015; Yamada et al., 2016). Despite this limitation, other studies have confirmed 100% specificity and positive predictive value (PPV) for CNPt-direct (Pasteran et al., 2015) and Carba NP tests (Tijet et al., 2013; Osterblad et al., 2014; CLSI, 2015).

Four isolates displayed elevated carbapenem MIC values but tested negative for carbapenemase production by both PCR and the NG-Test CARBA 5. This finding suggested non-carbapenemase-mediated carbapenem resistance, possibly involving efflux pump/porin loss coupled with the expression of ESBL/AmpC-type enzymes (Nordmann et al., 2012; Sekar et al., 2019). The NG-Test CARBA 5 showed excellent diagnostic performance for detecting all carbapenemases. Similar performance results have been observed in other single-center and multicenter evaluation studies of the NG-Test CARBA 5 in France and the United Kingdom (Boutal et al., 2018; Hopkins et al., 2018; Jenkins et al., 2020). To the authors’ knowledge, this study is the first to assess the performance of NG-Test CARBA 5 using isolates recovered from pets. The clinical applicability of the NG-Test CARBA 5 could be extended when applied directly to clinical samples. This approach simplifies testing, reduces the need for molecular methods, and may offer an effective means to streamline workflows and potentially reduce costs without affecting the overall quality of results (Jenkins et al., 2020).

The majority of CRE isolates were predominatly *K. pneumoniae* (84.8%). This finding aligns with the findings of Glaser et al. (2015), who reported that 83% of CRE cases during a suspected outbreak in Pennsylvania were attributed to *K. pneumoniae*. Also, carbapenemase resistant *K. pneumoniae* is one of the major pathogens causing high morbidity and mortality in Egyptian hospitals (Sherif et al., 2021; Gandor et al., 2022). Carbapenemase production is one of the primary mechanisms of *K. pneumoniae* carbapenem resistance, and the prevalence of carbapenem resistance genes has been rapidly changing (Wang et al., 2020). In line with the findings of a recent review indicating that OXA-48 and its variant, OXA-181, are among the most common in veterinary settings (Silva et al., 2022), our study revealed that *bla*_OXA-181_ was the most common gene found in CRE isolates (63.6%), followed by *bla*_IMP_ (60.6%), *bla*_OXA−48−like_ (43.6%), *bla*_KPC_ and *bla*_VIM_ (30.3% each), and *bla*_NDM_ (25.8%). The identified genotypes were *bla*_KPC-2_, *bla*_IMP-1_, *bla*_VIM-1_, *bla*_NDM-1_, and *bla*_NDM-5_. However, in dogs from India, the most common carbapenemase among GNB was NDM (52.3%) followed by OXA-181 (22.7%), KPC (18.2%), OXA-48 (13.6%), and VIM (4.6%) (Sankar et al., 2022). Furthermore, *bla*_OXA-181_ gene was identified in *E. coli* isolates from dogs and cats in Switzerland (Nigg et al., 2019) and Portugal (Brilhante et al., 2020). Fortunately, a single VIM-1-producing *K. pneumoniae* isolate (0.6%) was found in dogs in Madrid, Spain (González-Torralba et al., 2016). The presence of both OXA-181 and OXA-48 genes together was reported in *K. pneumoniae* and *Citrobacter freundii* isolates (Shanthi et al., 2013). The NDM-5-producing *E. coli* and NDM-1-producing *K. pneumoniae* have been identified from chicken meat in Egypt (Sadek et al., 2020). NDM-5-producing *E. coli* that shared some genetic features with human isolates was isolated from dogs in Egypt (Ramadan et al., 2020). *bla*_OXA-48_ and *bla*_OXA-181_ were found in ESBL-producing *E. coli* isolates from dairy cattle in Egypt (Braun et al., 2016). Human-to-animal transmission is also possible since *bla*_OXA-48_-, *bla*_KPC_,-and *bla*_NDM_-producing *K. pneumoniae* were isolated from hospitalized patients in Egypt (Gandor et al., 2022). MDR *K. pneumoniae* and *E. coli* isolates containing *bla*_NDM-1,_ *bla*_NDM-5_, *bla*_OXA-48_, *bla*_OXA-181_, and *bla*_KPC2_ are circulating in Egyptian hospitals (Soliman et al., 2020; Sherif et al., 2021). The frequent shift in the predominant carbapenemase genotype could be attributed to the introduction of strains harboring different carbapenemase genes from various regions or animals or the transfer of mobile elements carrying carbapenemase genes between species (Marques et al., 2019; Wang et al., 2020).

In the present study, our findings showed that 53% of the CRE isolates harboring at least one carbapenemase gene were from healthy pets. In Italy, 1.0% of non-hospitalized pets and 11.4% of hospitalized pets harbor carbapenem-resistant GNB (Gentilini et al., 2018). In Switzerland, Nigg et al. (2019) reported the carriage of carbapenem-resistant *E. coli* by pets (21.6%) that had acquired it during hospitalization and continued to carry it even after returning home, with only one dog (0.75%) being positive at hospital admission. Moreover, a high percentage of asymptomatic pet carriers of carbapenem-resistant *Acinetobacter baumannii* (2.7%) has been reported in France and highlighting pets as potential reservoirs for community-acquired infections (Hérivaux et al., 2016). Recently, surveillance conducted by emerging infection programs in eight regions of the United States from 2012 to 2015 revealed that 10% of CRE cases were community-acquired, affecting individuals without healthcare-associated risk factors (Bulens et al., 2023).

The antimicrobial resistance profiles of the CRE isolates revealed PDR (n = 20), XDR (n = 30), and MDR (n = 16) phenotypes, all of which were associated with the presence of at least one carbapenemase-encoding gene. This finding aligns with recent findings that carbapenem resistance in GNB, especially when carbapenemases are involved, is a primary driver of MDR and XDR phenotypes, often preceding the development of PDR (Meletis, 2016; Han et al., 2021). A more worrying finding is the complete resistance of our isolates to ceftaroline, a fifth-generation broad-spectrum anti-MRSA cephalosporin used to treat community-acquired pneumonia and complicated skin infections (El Hajj et al., 2017). Ceftaroline is approved only for *E. coli*, *K. pneumoniae*, and *K. oxytoca* (Magiorakos et al., 2012). This is concerning because infections caused by these high-priority CRE isolates pose treatment challenges and may exhibit resistance to various antibiotics, including critical antibiotics like carbapenems and 3^rd^ generation cephalosporins (W.H.O, 2017). Therefore, updated epidemiological data on AMR are crucial for selecting empirical treatment strategies, given the rapidly changing landscape of resistance patterns (Akova, 2016).

Notably, the infusion of high doses of carbapenem for longer duration has successfully treated CPE (MEM MIC up to 16 µg/mL) infections, which highlights the significance of MIC testing for the management of treatment (Morrill et al., 2015). Therefore, IPM or MEM could be considered viable options for CPE isolates, unless their MIC exceeds this threshold (Sekar et al., 2019). This information is clinically useful because in this study most CREs had MIC values ≤16 µg/mL. Moreover, CT and TIG retain their effectiveness against certain CRE isolates. Studies have demonstrated that combination therapy involving at least one carbapenem with CT or high-dose TIG or the use of aminoglycosides is more effective for treating CPE than carbapenem monotherapy, and even triple combinations have shown promise (Lee and Doi, 2014; Meletis, 2016; Jacobs et al., 2017).

## 5 Conclusions

The emergence of IMP, KPC, VIM, NDM, OXA-181, and OXA-48 CPE isolates with their PDR and XDR characteristics poses significant threats to public health. To manage CPE infection or colonization in pets, it is crucial to implement early detection methods for CPE, establish comprehensive surveillance systems, and enforce strict control measures for critical antibiotics.

## Data availability statement

The original contributions presented in the study are included in the article/**Supplementary Material**. Further inquiries can be directed to the corresponding authors.

## Ethics statement

The animal studies were approved by the Institutional Animal Care and Use Committee (IACUC) of Zagazig University, Egypt (Ref. No.: ZU-IACUC/2/F/416/2022). The studies were conducted in accordance with the local legislation and institutional requirements. Written informed consent was obtained from the owners for the participation of their animals in this study.

## Author contributions

YT: Conceptualization, Investigation, Methodology, Supervision, Writing – original draft, Writing – review & editing, Resources, Validation. AMA: Conceptualization, Investigation, Methodology, Resources, Supervision, Writing – review & editing. AA: Investigation, Resources, Supervision, Writing – review & editing. KH: Conceptualization, Data curation, Funding acquisition, Investigation, Methodology, Resources, Writing – review & editing. AS: Conceptualization, Investigation, Methodology, Resources, Validation, Writing – review & editing. SSE: Conceptualization, Investigation, Methodology, Resources, Validation, Writing – review & editing. ON: Data curation, Funding acquisition, Resources, Software, Validation, Writing – review & editing. IE: Data curation, Formal Analysis, Investigation, Software, Validation, Visualization, Writing – review & editing.
