# Supplementary material for: Emergence of pandrug-resistant carbapenemase-producing Enterobacterales in dogs and cats: a cross-sectional study in Egypt
Source: Front Cell Infect Microbiol. 2024 Mar 18;14:1318585. doi: 10.3389/fcimb.2024.1318585 (PMC10982511; doi:10.3389/fcimb.2024.1318585)
Supplement: Supplementary file 1 [file Table_1.docx]

Supplementary Material

Detection of pandrug-resistant carbapenemase-producing *Enterobacterales* from dogs and cats

**Yasmine H. Tartor^1^, Ahmed M. Ammar^1^, Adel Abdelkhalek^2^, Khlood A. Hassan^3^, Asmaa Shaker^4^, Shimaa S. Elnahriry^5^, Omid Nekouei^6*^, and Ibrahim Elsohaby^6,7,8*^**

^1^ Department of Microbiology, Faculty of Veterinary Medicine, Zagazig University, Zagazig, 44511, Egypt

^2^ Faculty of Veterinary Medicine, Badr University, Cairo 11829, Egypt

^3^ Veterinarian, Faculty of Veterinary Medicine, Zagazig University 44511, Zagazig, Egypt

^4^ Department of Microbiology, Veterinary Hospital, Faculty of Veterinary Medicine, University of Sadat City, Sadat City, Egypt

^5^Department of Bacteriology, Mycology and Immunology, Faculty of Veterinary Medicine, University of Sadat City, Menofia 32897, Egypt

^6^ Department of Infectious Diseases and Public Health, Jockey Club College of Veterinary Medicine and Life Sciences, City University of Hong Kong, Hong Kong SAR, China

^7^ Centre for Applied One Health Research and Policy Advice (OHRP), City University of Hong Kong, Hong Kong, Hong Kong SAR, China

^8^ Department of Animal Medicine, Faculty of Veterinary Medicine, Zagazig University, Zagazig 44511, Egypt

*** Correspondence:**Ibrahim Elsohaby ([ielsohab@cityu.edu.hk](mailto:ielsohab@cityu.edu.hk)) and Omid Nekouei ([omid.nekouei@cityu.edu.hk](mailto:omid.nekouei@cityu.edu.hk))

# Supplementary Figures and Tables

**Table 1S.** Demographic characteristics of the study population.

| **Parameter** | **Categories** | **No. (%) of animals** | | | | |
| --- | --- | --- | --- | --- | --- | --- |
|  |  | **Dogs (n = 62)** |  | **Cats (n = 48)** |  | **Total (n = 110)** |
| **Locality** |  |  |  |  |  |  |
|  | Cairo | 48 (77.4) |  | 12 (25) |  | 60 (54.6) |
|  | Dakahelia | 4 (6.6) |  | 36 (75) |  | 40 (36.4) |
|  | Sharkia | 10 (16.0) |  | -- |  | 10 (9.0) |
| **Gender** |  |  |  |  |  |  |
|  | Male | 26 (42.0) |  | 21 (43.8) |  | 47 (42.7) |
|  | Female | 25 (40.3) |  | 26 (54.2) |  | 51 (46.4) |
|  | Unknown | 11 (17.7) |  | 1 (2.0) |  | 12 (10.9) |
| **Breed** |  |  |  |  |  |  |
|  | Golden Retriever | 15 (24.2) |  | -- |  | 15 (16.6) |
|  | Rottweiler | 12 (19.4) |  | -- |  | 12 (10.9) |
|  | German Shepherd | 11 (17.4) |  | -- |  | 11 (10.0) |
|  | Griffon | 9 (14.5) |  | -- |  | 9 (8.2) |
|  | Husky | 5 (8.1) |  | -- |  | 5 (4.6) |
|  | Mongrel | 4 (6.5) |  | -- |  | 4 (3.6) |
|  | Pekingese | 2 (3.2) |  | -- |  | 2 (1.8) |
|  | Bullmastiff | 1 (1.6) |  | -- |  | 1 (0.9) |
|  | Cocker Spaniel | 1 (1.6) |  | -- |  | 1 (0.9) |
|  | Malibu | 1 (1.6) |  | -- |  | 1 (0.9) |
|  | Pit Bull | 1 (1.6) |  | -- |  | 1 (0.9) |
|  | Domestic short-haired | -- |  | 37 (77.1) |  | 37 (33.6) |
|  | Persian | -- |  | 11 (22.9) |  | 11 (10.0) |
| **Age** |  |  |  |  |  |  |
|  | Median (months) | 4 |  | 9 |  | 7 |
| **Stray** |  |  |  |  |  |  |
|  | Yes (Stray) | 4 (6.5) |  | 2 (4.2) |  | 6 (5.5) |
|  | No (domestic) | 58 (93.5) |  | 46 (95.8) |  | 104 (94.6) |
| **Health status** | |  |  |  |  |  |
|  | Healthy | 31 (50.0) |  | 20 (41.7) |  | 51 (46.4) |
|  | Diseased | 31 (50.0) |  | 28 (58.3) |  | 59 (53.6) |
| **Antibiotic use within 4 weeks** | |  |  |  |  |  |
|  | Yes | 16 (25.8) |  | 8 (16.7) |  | 24 (21.8) |
|  | No | 46 (74.2) |  | 40 (83.3) |  | 86 (78.2) |
| **Carbapenemase-producing *Enterobacterales*** | | |  |  |  |  |
|  | *E. coli* | 6 (9.7) |  | 7 (14.5) |  | 13 (11.8) |
|  | *K. pneumoniae* | 55 (88.7) |  | 38 (79.2) |  | 93 (84.6) |
|  | *K. oxytoca* | 1 (1.6) |  | 2 (4.2) |  | 3 (2.7) |
|  | No growth | 0 (0.0) |  | 1 (2.1) |  | 1 (0.9) |

**Table 2S.** Oligonucleotide primer sequences used in the study

| **Primer use and target gene** | **Primer name** | **Nucleotide sequence (5’→3’)** | **Amplicon size (bp)** | **Annealing temperature (**˚C) | **Reference** |
| --- | --- | --- | --- | --- | --- |
| ***K. pneumoniae* identification**  16S– 23S ITS* | Pf | ATTTGAAGAGGTTGCAAACGAT | 130 | 57 | (Liu et al., 2008) |
|  | Pr1 | TTCACTCTGAAGTTTTCTTGTGTTC |  |  |  |
| 1. ***K. oxytoca* identification** 2. *pehX* | PEH-C | GATACGGAGTATGCCTTTACGGTG | 344 | 59 | **(**Kovtunovych et al., 2003) |
|  | PEH-D | TAGCCTTTATCAAGCGGATACTGG |  |  |  |
| ***E. coli* identification**  *16S rRNA* | ECO-f | GACCTCGGTTTAGTTCACAGA | 585 | 56 | (Amit-Romach et al., 2004) |
|  | ECO-r | CACACGCTGACGCTGACCA |  |  |  |
| **Detection of carbapenemase genes**  *bla*_KPC_ | KPC-F | TGTTGCTGAAGGAGTTGGGC | 340 | 56 | (Mlynarcik et al., 2016) |
|  | KPC-R | ACGACGGCATAGTCATTTGC |  |  |  |
| *bla*_OXA-48_ | OXA-48A | TTGGTGGCATCGATTATCGG | 795 | 58 | (Poirel et al., 2004) |
|  | OXA-48B | GAGCACTTCTTTTGTGATGGC |  |  |  |
| *bla*_OXA-181_ | OXA181-F | ATGCGTGTATTAGCCTTATCG | 798 | 55 | (Mohanty et al., 2017) |
|  | OXA181-R | AACTACAAGCGCATCGAGCA |  |  |  |
| *bla*_VIM_ | VIM-F | AGTGGTGAGTATCCGACAG | 261 | 52 | (Sankar et al., 2022) |
|  | VIM-R | ATGAAAGTGCGTGGAGAC |  |  |  |
| *bla*_NDM_ | NDM-F | GGTTTGGCGATCTGGTTTTC | 621 | 52 | (Poirel et al., 2011) |
|  | NDM-R | CGGAATGGCTCATCACGATC |  |  |  |
| *bla*_IMP_ | IMP-F | TTG AAA AGCTTGATGAAG GCG | 615 | 60 | (Mohanty et al., 2017) |
|  | IMP-R | ACCGCCTGCTCTAATGTAAG |  |  |  |

*Internal transcribed spacer, bp: base pair.

**References:**

Amit-Romach, E., Sklan, D., and Uni, Z. (2004). Microflora Ecology of the Chicken Intestine Using 16S Ribosomal DNA Primers. *Poultry Science* 83**,** 1093-1098.

Kovtunovych, G., Lytvynenko, T., Negrutska, V., Lar, O., Brisse, S., and Kozyrovska, N. (2003). Identification of *Klebsiella oxytoca* using a specific PCR assay targeting the polygalacturonase pehX gene. *Res Microbiol* 154**,** 587-592.

Liu, Y., Liu, C., Zheng, W., Zhang, X., Yu, J., Gao, Q., Hou, Y., and Huang, X. (2008). PCR detection of Klebsiella pneumoniae in infant formula based on 16S–23S internal transcribed spacer. *International Journal of Food Microbiology* 125**,** 230-235.

Mlynarcik, P., Roderova, M., and Kolar, M. (2016). Primer Evaluation for PCR and its Application for Detection of Carbapenemases in *Enterobacteriaceae*. *Jundishapur J Microbiol* 9**,** e29314.

Mohanty, S., Mittal, G., and Gaind, R. (2017). Identification of Carbapenemase-mediated Resistance among Enterobacteriaceae Bloodstream Isolates: A Molecular Study from India. *Indian Journal of Medical Microbiology* 35**,** 421-425.

Poirel, L., Walsh, T.R., Cuvillier, V., and Nordmann, P. (2011). Multiplex PCR for detection of acquired carbapenemase genes. *Diagn Microbiol Infect Dis* 70**,** 119-123.

Poirel L, Héritier C, Tolün V, Nordmann P. 2004. Emergence of oxacillinase-mediated resistance to imipenem in *Klebsiella pneumoniae*. Antimicrob. Agents Chemother. 48:15–22

Sankar, S., Bosewell, A., and Mini, M. (2022). Molecular Detection of Carbapenem Resistant Gram Negative Bacterial Isolates from Dogs. *Indian journal of animal reserach* 56**,** 1557-1561.

**Table 3S:** Source, genotypes and accession numbers of 12 representative isolates

| **Isolate** | **Source** | **Genotype** | **Accession no** | **MIC (µg/mL) IMP** | **MIC (µg/mL) MEM** |
| --- | --- | --- | --- | --- | --- |
| ***E. coli*** | Dog | NDM5 | PP175343 | 8 | 2 |
|  | Dog | KPC2 | PP175342 | 8 | 2 |
|  | Dog | VIM-1 | PP175344 | 8 | 2 |
|  | Cat | IMP-1 | PP158752 | 8 | 2 |
|  | Dog | OXA-48 | PP158753 | 4 | 2 |
|  | Dog | OXA-181 | PP158749 | 4 | 2 |
| ***K. pneumoniae*** | Cat | NDM1 | PP158750 | 16 | 4 |
|  | Dog | KPC2 | PP175341 | 16 | 2 |
|  | Cat | VIM-1 | PP158751 | 8 | 128 |
|  | Cat | IMP-1 | PP175345 | 8 | 2 |
|  | Cat | OXA-48 | PP175346 | 4 | 2 |
|  | Cat | OXA-181 | PP175347 | 4 | 2 |

**Table 4S:** Comparison of the performance of NG-Test CARBA 5 with PCR for detection of carbapenem-resistant *Enterobacteriales*

| **Isolate no.** | **Isolate code** | **Species** | **Carbapenemase genes** | ***NG-Test CARBA 5** |
| --- | --- | --- | --- | --- |
| 1 | D2 | *K. pneumoniae* | KPC (+ve)  OXA-48 (+ve)  OXA-181 (+ve)  NDM (+ve)  VIM (+ve)  IMP (+ve) | KPC (+ve)  OXA (+ve)  NDM (+ve)  VIM (+ve)  IMP (+ve) |
| 2 | D4 | *K. pneumoniae* | KPC (+ve)  OXA-48 (+ve)  OXA-181 (+ve)  NDM (-ve)  VIM (-ve)  IMP (-ve) | KPC (+ve)  OXA (+ve)  NDM (-ve)  VIM (-ve)  IMP (-ve) |
| 3 | D6 | *E. coli* | KPC (+ve)  OXA-48 (+ve)  OXA-181 (+ve)  NDM (+ve)  VIM (+ve)  IMP (+ve) | KPC (+ve)  OXA (+ve)  NDM (+ve)  VIM (+ve)  IMP (+ve) |
| 4 | D12 | *E. coli* | KPC (-ve)  OXA-48 (-ve)  OXA-181 (+ve)  NDM (-ve)  VIM (-ve)  IMP(+ve) | KPC (-ve)  OXA (+ve)  NDM (-ve)  VIM (-ve)  IMP (+ve) |
| 5 | D1 | *K. pneumoniae* | **KPC** (-ve)  OXA-48 (+ve)  OXA-181 (+ve)  NDM (-ve)  **VIM** (-ve)  IMP (+ve) | KPC (+ve)  OXA (+ve)  NDM (-ve)  VIM (+ve)  IMP (+ve) |
| 6 | D8 | *K. pneumoniae* | KPC (-ve)  OXA-48 (-ve)  **OXA-181** (+ve)  NDM (+ve)  VIM (-ve)  IMP (-ve) | KPC (-ve)  **OXA (-ve)**  NDM (+ve)  VIM (-ve)  IMP (-ve) |
| 7 | D11 | *K. pneumoniae* | KPC (+ve)  OXA-48 (-ve)  OXA-181 (-ve)  NDM (-ve)  VIM (-ve)  IMP (-ve) | KPC (+ve)  OXA (-ve)  NDM (-ve)  VIM (-ve)  IMP (-ve) |
| 8 | D12 | *K. pneumoniae* | KPC (-ve)  OXA-48 (-ve)  OXA-181 (-ve)  NDM (-ve)  VIM (-ve)  IMP (+ve) | KPC (-ve)  OXA (-ve)  NDM (-ve)  VIM (-ve)  IMP (+ve) |
| 9 | D14 | *K. pneumoniae* | KPC (-ve)  OXA-48 (+ve)  OXA-181 (-ve)  NDM (-ve)  VIM (+ve)  **IMP** (+ve) | KPC (-ve)  OXA (+ve)  NDM (-ve)  VIM (+ve)  **IMP (-ve)** |
| 10 | D15 | *K. pneumoniae* | KPC (-ve)  OXA-48 (+ve)  OXA-181 (-ve)  NDM (-ve)  VIM (-ve)  IMP (-ve) | KPC (-ve)  OXA (+ve)  NDM (-ve)  VIM (-ve)  IMP (-ve) |
| 11 | D22 | *K. pneumoniae* | KPC (-ve)  OXA-48 (+ve)  OXA-181 (+ve)  NDM (-ve)  VIM (+ve)  IMP (-ve) | KPC (-ve)  OXA (+ve)  NDM (-ve)  VIM (+ve)  IMP (-ve) |
| 12 | D23 | *K. pneumoniae* | KPC (+ve)  OXA-48 (-ve)  OXA-181 (+ve)  NDM (-ve)  **VIM** (-ve)  IMP (-ve) | KPC (+ve)  OXA (+ve)  NDM (-ve)  VIM (+ve)  IMP (-ve) |
| 13 | C26 | *K. pneumoniae* | KPC (+ve)  OXA-48 (+ve)  OXA-181 (+ve)  NDM (-ve)  VIM (+ve)  IMP (+ve) | KPC (+ve)  OXA (+ve)  NDM (-ve)  VIM (+ve)  IMP (+ve) |
| 14 | D29 | *K. pneumoniae* | KPC (-ve)  OXA-48 (-ve)  OXA-181 (-ve)  NDM (-ve)  VIM (-ve)  IMP (-ve) | KPC (-ve)  OXA (-ve)  NDM (-ve)  VIM (-ve)  IMP (-ve) |
| 15 | C5 | *K. pneumoniae* | KPC (-ve)  OXA-48 (-ve)  OXA-181 (-ve)  NDM (-ve)  VIM (-ve)  IMP (-ve) | KPC (-ve)  OXA (-ve)  NDM (-ve)  VIM (-ve)  IMP (-ve) |
| 16 | C17 | *E. coli* | KPC (+ve)  OXA-48 (+ve)  OXA-181 (+ve)  NDM (+ve)  VIM (-ve)  IMP (-ve) | KPC (+ve)  OXA (+ve)  NDM (+ve)  VIM (-ve)  IMP (-ve) |
| 17 | C24 | *K. pneumoniae* | KPC (-ve)  OXA-48 (-ve)  OXA-181 (-ve)  NDM (-ve)  VIM (-ve)  IMP (-ve) | KPC (-ve)  OXA (-ve)  NDM (-ve)  VIM (-ve)  IMP (-ve) |
| 18 | C25 | *K. pneumoniae* | KPC (-ve)  OXA-48 (-ve)  OXA-181 (-ve)  NDM (-ve)  VIM (-ve)  IMP (-ve) | KPC (-ve)  OXA (-ve)  NDM (-ve)  VIM (-ve)  IMP (-ve) |
| 19 | C26 | *K. oxytoca* | KPC (+ve)  OXA-48 (+ve)  OXA-181 (-ve)  NDM (+ve)  VIM (-ve)  IMP (+ve) | KPC (+ve)  OXA (+ve)  NDM (+ve)  VIM (-ve)  IMP (+ve) |
| 20 | D10 | *K. pneumoniae* | KPC (-ve)  OXA-48 (-ve)  OXA-181 (+ve)  NDM (-ve)  VIM (-ve)  **IMP** (+ve) | KPC (-ve)  OXA (+ve)  NDM (-ve)  VIM (-ve)  **IMP (-ve)** |

* In NG CARBA 5 results the underlined genes were false-positive and those in boldface type were false-negative.
